# The chloroplast genome structure of *Turpinia affinis* (Staphyleaceae)

**DOI:** 10.1080/23802359.2024.2392750

**Published:** 2024-08-19

**Authors:** Mei Zhang, Shun Yu, Roujun Wang, Shibiao Pu

**Affiliations:** a College of Traditional Chinese Medicine, Nanjing University of Chinese Medicine, Nanjing, China; b Yunnan Institute of Traditional Chinese Medicine and Material Medical, Kumning, China; c College of Traditional Chinese Medicine, Yunnan University of Chinese Medicine, Kunming, China; d Department of Diabetes and Endocrinology, Kunming Municipal Hospital of Traditional Chinese Medicine, Kunming, China

**Keywords:** Chloroplast genome, *Turpinia affinis*, Turpiniae Folium, substitute

## Abstract

The species *Turpinia affinis* Merr. et Perry 1941 is widely distributed throughout southwestern China. In folk medicine, this species is often used as a substitute for the Chinese medicine Turpiniae Folium, whose legal origin is *T. arguta* (Lindl.) Seem. In order to ascertain the relationship between these two species, the chloroplast genome of *T. affinis* was aequenced and assembled, resulting in a typical quadripartite molecule with a length of 160,769 base pairs and an overall GC content of 37.3%. Additionally, 131 genes were annotated, comprising 86 protein-coding genes, 37 tRNA genes, and 8 rRNA genes. A maximum likelihood analysis demonstrated that the *Turpinia* species form a monophyletic clade, with *T. affinis* positioned as the sister taxon to the clade comprising the remaining species within the genus. This outcome enhances the genomic data for the genus *Turpinia* and will contribute to further investigations into phylogenetics, evolution, and sustainable resource utilization.

## Introduction

*Turpinia* Vent. represents the largest genus within the Staphyleaceae family, encompassing over 30 species distributed across the globe (Li et al. 2008). A number of species within the genus are commonly used as medicinal herbs in traditional folk medicine for the treatment of pharyngitis, amygdalitis, and tonsillar abscess. Some of the most frequently utilized species include *Turpinia affinis* Merr. et Perry 1941, *T. montana* (Bl.) Kurz., and *T. arguta* (Lindl.) Seem (Editorial Committee of Zhonghua Bencao 1999; Lei et al. 2019). Nevertheless, only one species, *T. arguta*, is officially documented in the Pharmacopeia of the People’s Republic of China 2020 (Chinese edition) as the legal source of the Chinese medicine, Turpiniae Folium (China Pharmacopoeia Committee 2020). In folk medicine, *T. affinis* is frequently employed as a substitute for Turpiniae Folium and can occasionally be found in its dried medicinal herbs. To enhance our genetic comprehension of *Turpinia* and clarify the relationship between *T. affinis* and *T. arguta*, we characterized and reported the complete plastid genome sequence of *T. affinis* in this study. This genetic information will prove invaluable for molecular identification and authentication of medicinal plants of the genus *Turpinia*.

## Materials and methods

**Figure 1.**
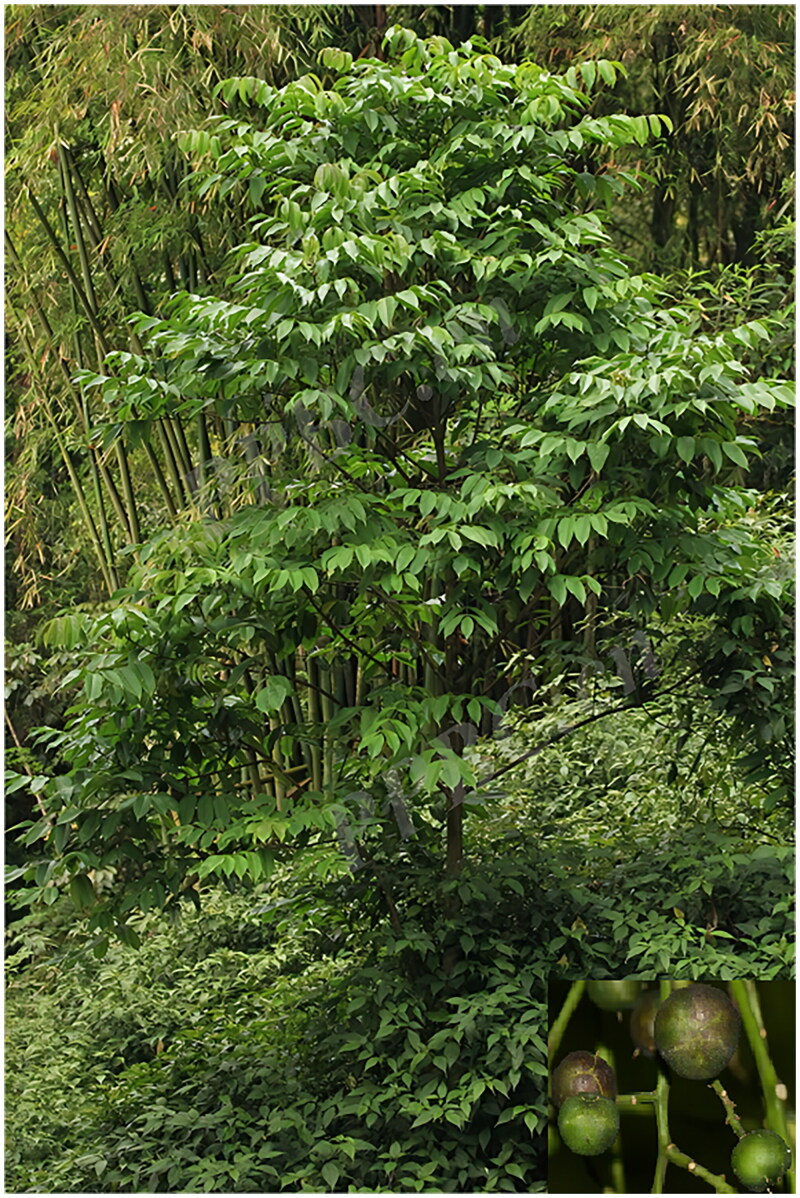
The analyzed sample of *Turpinia affinis* Merr. et Perry. The photograph was taken by shibiao Pu in Kunming Botanic Garden, Kunming, China. Main identifying traits: Leaves odd-pinnate, leaflets 2–5, base cuneate to obtuse, margin crenate, apex acuminate; panicle ca. 30 cm; berry subglobose, 1–1.5 cm in diam.

The fresh leaves of *Turpinia affinis* were collected from the Kunming Botanic Garden in Yunnan Province (25.14 N, 102.74E) (Figure 1). The voucher specimen (Pu018, managed by Shaotian Chen, email: chenshaotian@ynutcm.edu.cn) was deposited in the Museum of Ethnic Medicine, Yunnan University of Chinese Medicine. The total DNA was extracted from silica gel dried leaves using a modified CTAB (cetyl trimethylammonium bromide) method (Doyle and Doyle 1987). The fragmented genomic DNA was used to construct the short-insert (300–500 bp) libraries according to the manufacturer’s manual (Illumina), and then sequenced on an Illumina Hiseg 2000 platform. The filtered sequencing data were used to assemble the chloroplast genome sequence with NOVOPlasty (Dierckxsens et al. 2017), and the cp genome was annotated using the GeSeq platform (https://chlorobox.mpimp-golm.mpg.de/geseq.html) (Tillich et al. 2017), with reference to the plastome of *T. arguta* (Lindl.) Seem (Accession no.: MT859133) (Cao et al. 2020). To confirm the accuracy of the assembly, the clean reads were mapped to the assembled chloroplast genome to evaluate the coverage depth using BWA (Li and Durbin 2009). Additionally, the arrangements of genes containing introns were examined using the CPGView web platform (http://www.1kmpg.cn/cpgview, Liu et al. 2023).

To further investigate the phylogenetic position of *Turpinia affinis*, we constructed a phylogenetic tree based on 14 complete chloroplast genome sequences relevant to Staphyleaceae, with *Laguncularia racemosa* C.F.Gaertn (MK726017) serving as the outgroup. These 14 sequences were aligned using the MAFFT version 7 software (Katoh et al. 2002). The maximum likelihood (ML) tree was constructed using IQ-TREE v2.0.4 (Nguyen et al. 2015). The K3Pu + F+R4 model was identified as the best-fit model according to Bayesian information criterion using ModelFinder (Kalyaanamoorthy et al. 2017). The ultrafast bootstrap (UFBoot) approach was employed to assess branch supports with 1,000 replicates (Hoang et al. 2018).

## Result

**Figure 2.**
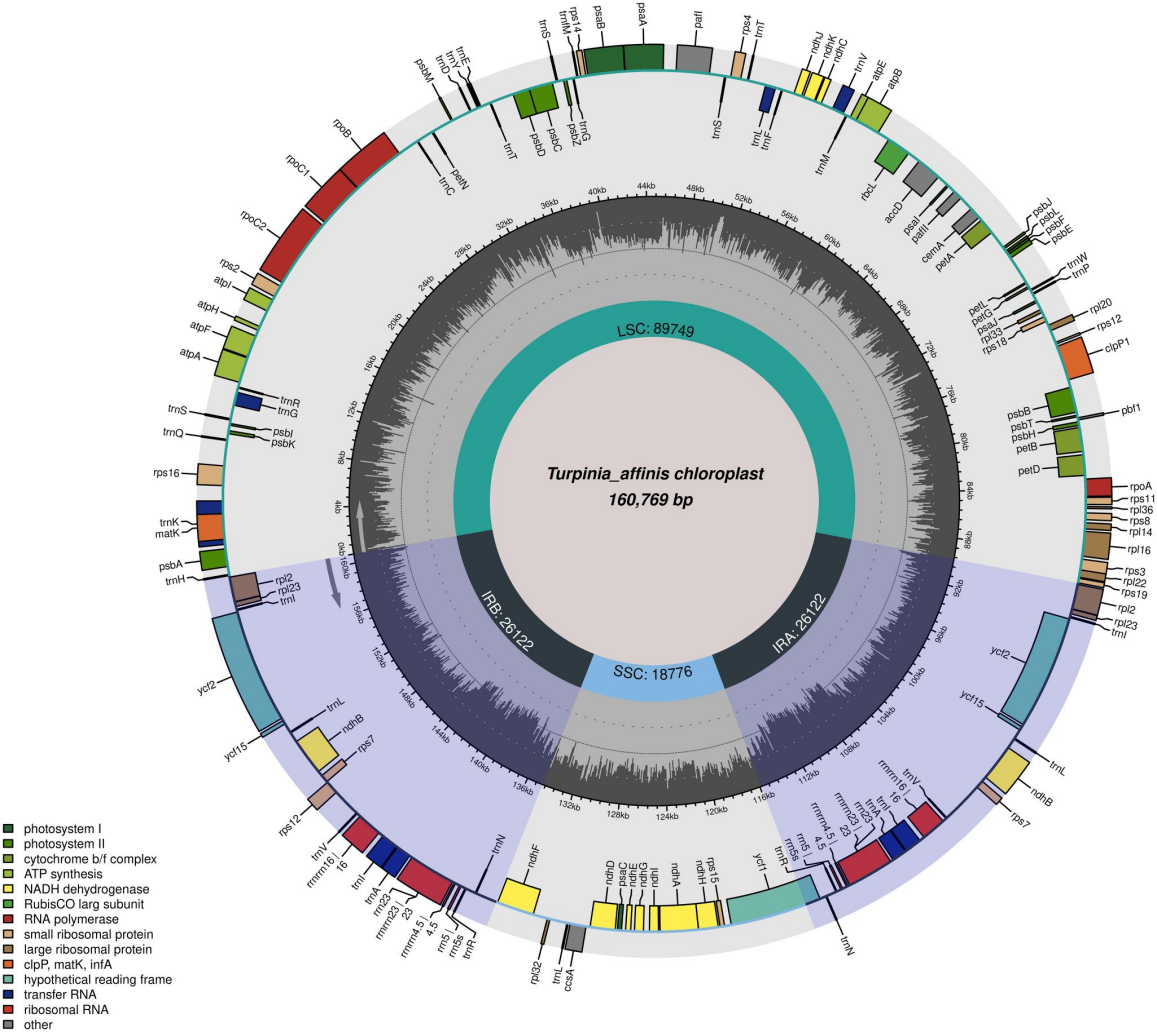
The circular map of *Turpinia affinis* Merr. et Perry chloroplast genome. In the chloroplast genome, the small single-copy (SSC) and large single-copy (LSC) regions are separated by inverted repeats (IRs: IRA and IRB). Genes on the inside of the circle are transcribed in a clockwise direction and genes on the outside of the circle are transcribed in a counter-clockwise direction. The small gray bar graphs inner circle shows the GC contents. Genes are color-coded by their functional classification. The functional classification of the genes is shown on the left bottom.

The cp genome assembly produced a classic quadripartite molecule, exhibiting a length of 160,769 base pairs (bps) and an overall GC content of 37.3%. The average read coverage depth for the assembled cp genome was ×1725.6 (Supplementary Figure S1). This molecule can be subdivided into the large single copy (LSC), the short single copy (SSC), and two inverted regions (IR) (Figure 2). The length of LSC, SSC, and IR regions are 89,749 bps, 18,776 bps, and 26,122 bps, respectively, and their respective GC contents are 35.5%, 31.7%, and 42.7%. The plastome of *Turpinia affinis* comprises 131 genes, including 86 protein-coding genes, 37 tRNA genes, and 8 rRNA genes. A total of 12 cis-splicing genes were detected among the coding sequences (CDS) (Supplementary Figure S2A). Of the cis-splicing genes, 10 were found to contain one intron, including rps16, atpF, rpoC1, petB, petD, rpl16, rpl2, ndhB, ndhA, and rpl2, while. Two genes, paf1 and clpP1, were observed to contain two introns. Additionally, the gene structure of the trans-splicing gene rps12 was also identified (Supplementary Figure S2B). The assembled and annotated cp genome have been submitted to GenBank by Yunhui Jiang and Shaotian Chen (Accession no.: OQ909073).

**Figure 3.**
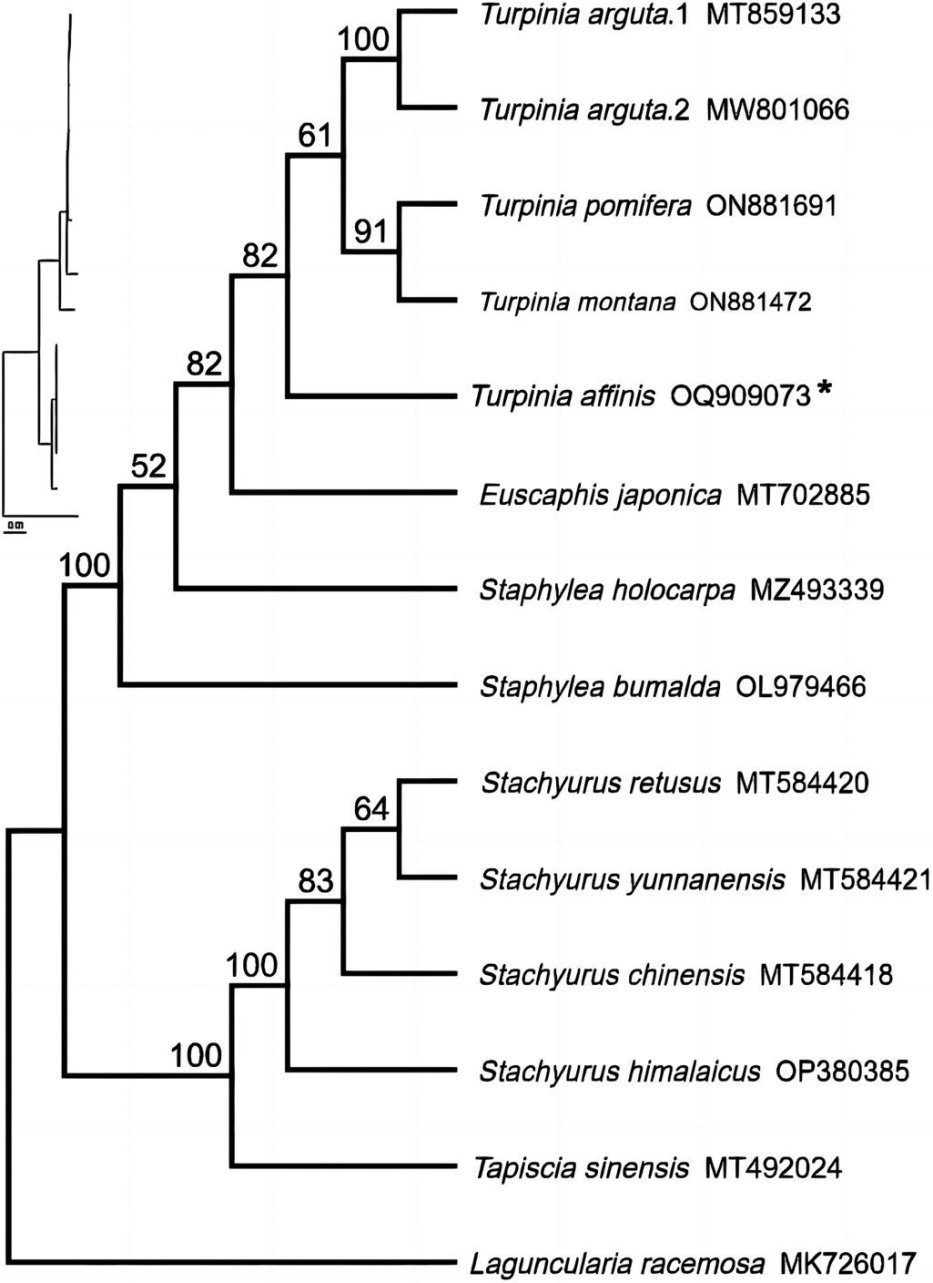
The maximum-likelihood phylogeny of *Turpinia affinis* and its close relatives using whole genome sequences. The bootstrap values based on 1,000 replicates were shown on each node in the cladogram tree. The sequences used for tree construction are as follows: *T. arguta* (MT859133, MW801066) (Cao et al. 2020), *T. pomifera* (ON881691), *T. montana* (ON881472), *T. affinis* (OQ909073), *Euscaphis japonica* (MT702885), *staphylea holocarpa* (MZ493339), *S. bumalda* (OL979466), *S. retusus* (MT584420), *S. yunnanensis* (MT584421), *S. chinensis* (MT584418), *S. himalaicus* (OP380385), *Tapiscia sinensis* (MT492024), *Laguncularia racemosa* (MK726017).

The results of the phylogenetic analysis showed that the four species of the *Turpinia* genus constituted a monophyletic group, with *T. affinis* positioned as the sister to the clade of the remaining three species of the genus (Figure 3). The Staphyleaceae family constituted a clade that included three genera: *Turpinia*, *Euscaphis*, and *Staphylea*. The other clade was composed of two genera: *Tapiscia* and *Stachyurus* from the Tapisciaceae and Stachyuraceae families, respectively.

## Discussion and conclusion

Turpiniae Folium is a traditional Chinese medicine that has been in use for centuries. It is composed exclusively of the desiccated leaves of the *Turpinia arguta* plant. The 2020 edition of the Pharmacopeia of the People’s Republic of China (in Chinese) explicitly designates *T. arguta* as the sole authorized botanical source for this herb medicine. However, within the domain of folk medicine, a notable divergence from this official practice is evident, where the desiccated leaves of *T. affinis* are frequently utilized as an alternative to Turpiniae Folium and are often found combined with the crude drugs derived from *T. arguta*.

Our comprehensive analysis has revealed a clear phylogenetic divergence between *T. affinis* and *T. arguta*, indicating that they represent distinct evolutionary lineages. Moreover, the two species display notable differences on morphology, particularly in their phyllotaxy patterns and fruit size. It is therefore imperative to conduct further inquiries into the chemical constituents and consistency assessments of these two medicinal herbs, with the aim of ascertaining the suitability of the dried leaves of *T. affinis* as a viable substitute for Turpiniae Folium.

Furthermore, our study introduces a pioneering primary characterization of the chloroplast genome of *T. affinis*. This contribution not only augments our understanding of this species but also establishes a robust foundation for future research endeavors encompassing molecular identification, evolutionary biology, and conservation strategies pertaining to the entire genus.

## Supplementary Material

Supplementary Figure S1.pdf

Supplementary Figure S2.pdf

## Data Availability

The data that support the findings of this study are openly available in GenBank of NCBI at https://www.ncbi.nlm.nih.gov/nuccore/OQ909073. The associated BioProject, GSA and Bio-Sample numbers in National Genomics Data Center (https://bigd.big.ac.cn/gsa/) are PRJCA016656, CRA010892, and SAMC1234185, respectively.
