# Supplementary material for: The chloroplast genome structure of Turpinia affinis (Staphyleaceae)
Source: Mitochondrial DNA B Resour. 2024 Aug 19;9(8):1103–6. doi: 10.1080/23802359.2024.2392750 (PMC11334737; doi:10.1080/23802359.2024.2392750)
Supplement: Supplementary Figure S2.pdf [file TMDN_A_2392750_SM1803.pdf]

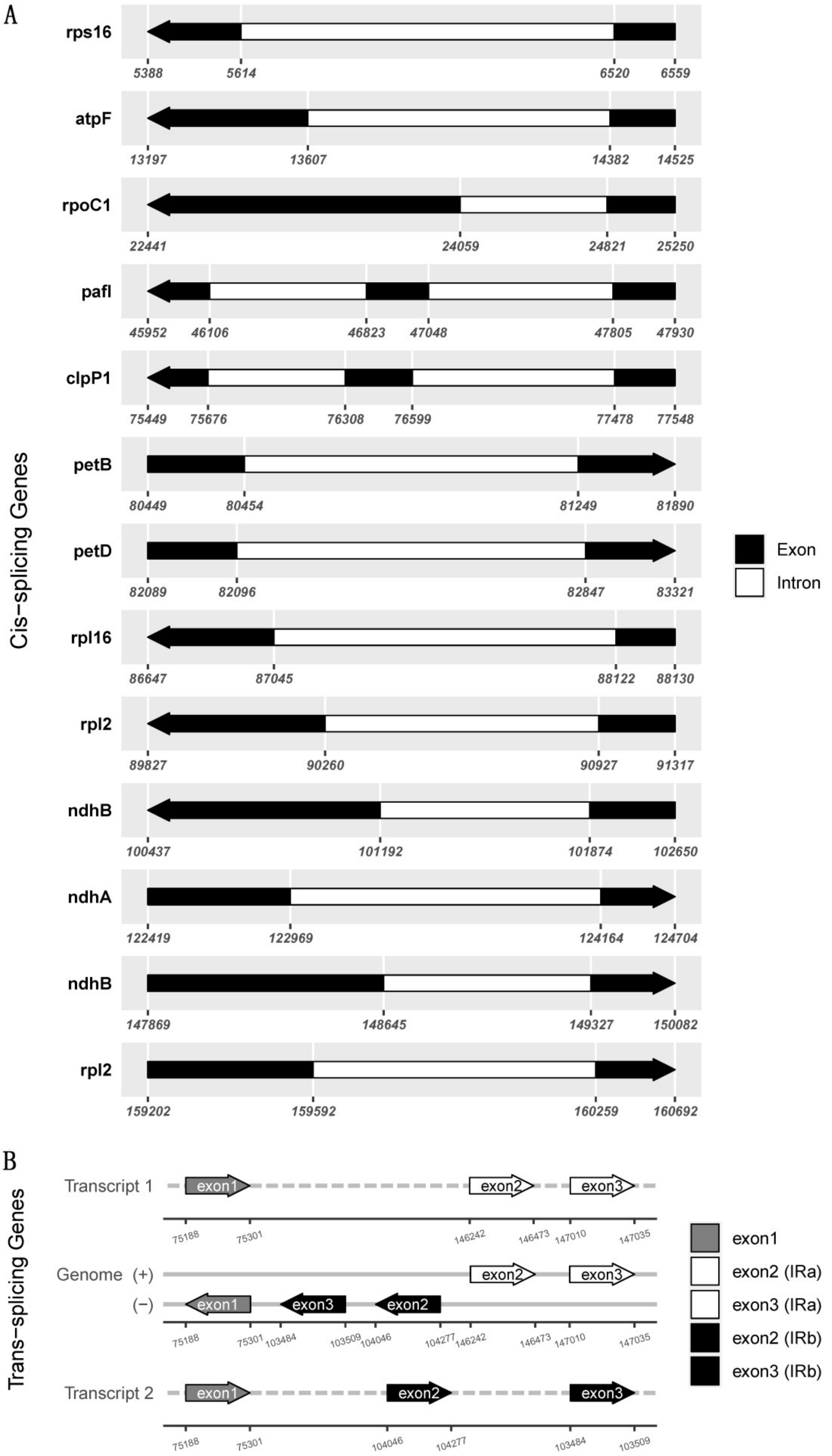

Supplementary Figure S2. Schematic map of the cis (A) and trans (B) splicing genes in the chloroplast genome of *Turpinia affinis* (OQ909073).
